# *In Vitro* characterization of narciclasine: solubility, metabolic stability, and P-glycoprotein substrate status

**DOI:** 10.3389/fphar.2026.1768477

**Published:** 2026-04-28

**Authors:** Ravi Akkireddy, In-Hyoung Yang, Srinivas Lenkalapelly, Ganesh J. Kshirsagar, Harry A. May, Min H. Kang

**Affiliations:** 1 Cancer Center, School of Medicine, Texas Tech University Health Sciences Center, Lubbock, TX, United States; 2 Department of Pediatrics, School of Medicine, Texas Tech University Health Sciences Center, Lubbock, TX, United States; 3 Graduate School of Biomedical Sciences, Texas Tech University Health Sciences Center, Lubbock, TX, United States

**Keywords:** ADME, metabolism, narciclasine, p-glycoprotein, stability, solubility, permeability

## Abstract

**Background:**

Narciclasine has demonstrated anticancer activity at low nanomolar concentrations in various preclinical cancer models, but no clinical data in cancer patients are available. Although its biological activity and structure-activity relationships are relatively well characterized, the pharmacological properties of narciclasine have not been reported. This information will benefit the research to further develop narciclasine. The goal of the current study is to characterize the physicochemical properties, metabolic stability, and P-glycoprotein substrate status of narciclasine.

**Methods:**

We first developed a robust HPLC-tandem mass spectrometry assay to measure narciclasine in mouse plasma. Then, we assessed the stability, metabolic pathways, and cytochrome P450 inhibitory effect of narciclasine. We also evaluated narciclasine for its P-glycoprotein substrate status using two methods: 1) Caco-2 permeability assay, and 2) cytotoxicity assay in human cancer cells with exogenous expression of P-glycoprotein.

**Results:**

Narciclasine was stable in the plasma of the four species tested. It showed metabolic stability in human liver microsomes and hepatocytes, but in other species, metabolic clearance was higher than in humans, indicating that humans may metabolize narciclasine minimally. Further studies to identify the metabolic pathway of narciclasine detected trace amounts of reduction and glucuronidation in human liver microsomes. Narciclasine was highly soluble under both thermodynamic and kinetic conditions over a range of pH values, with a lipophilicity value of 0.40, indicating it is hydrophilic. P-glycoprotein (P-gp) overexpression reduced the *in vitro* cytotoxicity of narciclasine less than that of another known P-gp substrate, vincristine.

**Conclusion:**

Narciclasine demonstrates metabolic stability in human liver microsomes and hepatocyts, and its cancer cell penetration is minimally affected by P-glycoprotein. These data will inform future development of narciclasine as a cancer therapeutic agent.

## 1 Introduction

Narciclasine is an isoquinoline alkaloid isolated from narcissus bulbs in 1967 and is also known as lycoricidinol (Fürst, 2016). Studies have shown that narciclasine reduces inflammation in animals with sepsis and suppresses cytokine production, resulting in attenuated sepsis-induced myocardial injury (Kingsley et al., 2020; Tang et al., 2021). In cancer studies, narciclasine has been shown to induce cell cycle arrest and inhibit GTPase and kinase activities, including Rho kinase and serine-threonine kinases (Kornienko and Evidente, 2008; Bräutigam et al., 2019; Qiu et al., 2022; Glotfelty et al., 2023). The compound has demonstrated *in vitro* or *in vivo* xenograft activities against multiple malignancies, including brain cancer, endometrial cancer, lymphoma, gastric cancer, and small cell lung cancer (Ingrassia et al., 2008; Matveenko et al., 2009; Van Goietsenoven et al., 2013; Fürst, 2016; Gopalakrishnan et al., 2020; Yuan et al., 2021; Wei et al., 2023).

The diverse biological activities of narciclasine are attributed to multiple mechanisms of action. Its anticancer effects are thought to arise from a combination of processes, including inhibition of protein biosynthesis, activation of GTPase RhoA, inhibition of RUNX1 and STAT3, interference with DNA-PKcs, induction of autophagy, and inhibition of eEF1A and topoisomerase I (Van Goietsenoven et al., 2013; Bräutigam et al., 2019; Mill et al., 2019; Yuan et al., 2021; Lv et al., 2022; Wang et al., 2023; Wei et al., 2023). Beyond oncology, narciclasine has been reported to inhibit phospholipase 2 and downregulate endothelial TNF receptor 1, both of which are implicated in psoriasis-like dermatitis and systemic inflammation (Stark et al., 2019; Kong et al., 2022). A systematic investigation into additional molecular targets of narciclasine would deepen our mechanistic understanding of the compound and support its further development as an anticancer therapeutic.

Despite a low nanomolar *in vitro* cytotoxicity across various cancer cell lines and a reasonable therapeutic window between cancer and non-cancerous cells (Wei et al., 2023), narciclasine has demonstrated only modest *in vivo* anticancer activity in xenograft mouse models, which may have hindered its advancement into human clinical trials. ADME and toxicology properties—encompassing absorption, distribution, metabolism, excretion, and toxicity—are critical determinants of a drug candidate’s clinical success. Nearly half of all drug failures are attributable to inadequate efficacy, frequently driven by poor bioavailability resulting from suboptimal intestinal absorption or rapid metabolism (Li, 2001). Many such challenges can be traced to deficiencies in ADME, pharmacokinetics (PK), toxicokinetics (TK), and formulation properties (Caldwell and Yan, 2014).

Given the absence of prior reports on the ADME properties and metabolite profiles of narciclasine, we developed novel UPLC-MS/MS and UPLC methods for the quantification of narciclasine and its metabolites in plasma and multiple biological matrices (Svagrová et al., 1991; Chen et al., 2021). We then conducted a comprehensive investigation of the compound’s ADME characteristics and assessed whether narciclasine functions as a substrate of P-glycoprotein (P-gp), a common property of natural products. To our knowledge, this study is the first to characterize the pharmacological properties of narciclasine, and the findings reported here provide a valuable foundation for its future development.

## 2 Materials and methods

### 2.1 Reagents and chemicals

Narciclasine was synthesized by Aktin Chemicals, Inc. (China). The reagents and materials used for the current project are listed in Supplementary Table S1.

### 2.2 Sample preparation

Samples for assay validation were processed using a protein precipitation method with acetonitrile. 20 μL of sample was treated with 200 µL of internal standard (Clofarabine, final concentration 20 ng/mL), vortexed for 5 min, and centrifuged for 10 min at 3300 g. Fifty µL of supernatant was diluted with 200 µL of Milli-Q water in a 96-deep-well plate. Precision and accuracy were assessed within a single run (intra-run) and across four consecutive days (inter-run) at four QC levels (LLOQ, low, medium, and high), with six replicates per level. Data were deemed acceptable if accuracy was within ±15% of nominal values and precision (RSD) was within ±15%, except for the LLOQ, which allowed for precision up to ±20%. To quantify extraction recovery and matrix effects, six replicates of three QC concentrations (low, medium, high) and six replicates of the IS at 20 ng/mL were analyzed for narciclasine. Extraction recovery was calculated by comparing peak areas of processed spiked plasma samples to those of standards in the mobile phase.

### 2.3 Liquid chromatography and mass spectrometry conditions

A sensitive and selective LC-MS/MS method was developed to quantify narciclasine in plasma and other matrices. The analytes were separated by chromatography on a Shimadzu LC-40 (KYOTO, Japan) system. The analytical column was an XBridge C18 3.5 µm 2.1 × 50 mm (Waters Corporation, Milford, MA), and the column oven temperature was maintained at 35 °C. The mobile phases consisted of 3 mM ammonium acetate in water (A) and 100% acetonitrile (B), and were delivered under gradient elution conditions. The HPLC gradient program was as follows: 95% aqueous/5% organic for 0.0–0.1 min, then a linear gradient to 5% aqueous/95% organic at 1.0 min, hold for 1.5 min, then bring back to 95% aqueous/5% organic at 1.6 min, hold the same up to 2.5 min. Flow rate of 0.4 mL/min. Needle wash of 10:50:40: IPA/Acetonitrile/Water was also instituted to reduce carryover in subsequent runs. The column eluent within the 0.2–2.2 min time window was diverted to a mass spectrometer using a switching valve (Valco Instruments Co. Inc., Houston, TX, United States). The autosampler temperature was kept at 8 °C, and the injection volume was 1.0 μL.

The SCIEX 6500+ QTRAP mass spectrometer (Framingham, MA) was operated in positive electrospray ionization (ESI) mode, and the analysis was performed via multiple reaction monitoring (MRM). The source parameters were as follows: ion spray voltage, 5500 V; capillary temperature, 600 °C; curtain gas (CUR), nebulizer gas (GS1), and turbo gas (GS2) pressures, 38, 60, and 65 psi, respectively; collision-activation dissociation (CAD), medium. The precursor to product ion transitions was as follows: m/z 308.1 → 248.0 for narciclasine, with the declustering potential (DP), collision energy (CE), and collision cell exit potential (CXP) set to 100, 55 and 12 V, respectively; m/z 304.2 → 170.0 for Clofarabine with the DP, CE and CXP set to 100, 29 and 12 V, respectively.

### 2.4 Kinetic solubility

Kinetic solubility was determined (Lin and Pease, 2016; Sou and Bergström, 2018) using 50 mM DMSO stock solutions, prepared by weighing 15 mg of the solid sample into a 2 mL vial and then adding ∼1 mL DMSO to obtain a final concentration of 50 mM. The stock solutions were checked for precipitation. We used 50 mM DMSO stock solutions, fully dissolved, for aqueous solubility assessment. The detailed procedures of the experiments are described in Supplemental Methods.

### 2.5 Lipophilicity

We performed the LogD7.4 assay, the logarithm of the n-octanol/water distribution coefficient measured at pH 7.4, using the standard shake flask method (Rutkowska et al., 2013; Andrés et al., 2015) to determine the lipophilicity of narciclasine. Five hundred µL of saturated octanol (both solutions should be saturated before the compound introduction, buffer with n-octanol and n-octanol with buffer) was added to a 2.0 mL vial, followed by 10 µL of the test compound (10 mM). The contents were mixed for 30 s, then the pre-saturated buffer was added. The vial was then vortexed at 1,200 rpm for 1 h at room temperature. After 20 min of equilibration, the mixture was centrifuged at 4,000 rpm for 30 min. The buffer and octanol layers were then separated and analyzed using HPLC. The lipophilicity and solubility experiments used the same analytical conditions described under Supplemental Methods.

Calculations:

$$\log D=\log\;\left(Octanol\;Area/buffer\;area\right)$$

### 2.6 Plasma stability

We then measured the *in vitro* stability of the narciclasine in the plasma of mice, humans, rats, and beagle dogs. The experiment was performed as follows: plasma was prepared, and the reactions were initiated by adding 4 µL of 100 μM test compound to 396 µL of preincubated plasma, resulting in a final concentration of 1 μM (Hartman, 2003; Di et al., 2005). The detailed conditions for plasma stability are included in the Supplemental Methods.

An assessment of dilution integrity, ensuring accurate measurement above the ULOQ, was performed by diluting samples spiked at 32 ng/mL (5-fold) and 16 ng/mL (10-fold) with the blank matrix. Accuracy and precision were determined from the six replicates per dilution.

### 2.7 Metabolic stability and inhibition of metabolism

Metabolic stability experiments were conducted using liver microsomes and hepatocytes from different species, as previously discussed (Lundquist et al., 2014; Shah et al., 2016; Joshi et al., 2025). The detailed procedures of the experiments are described in Supplemental Methods.

### 2.8 Plasma protein binding using RED

Plasma protein binding experiments were carried out in male human K2EDTA plasma, male CD-1 plasma, beagle dog plasma, and SD rat plasma (innovative) using a Pierce Rapid Equilibration Dialysis (RED) device. Briefly, plasma (350 μL) containing narciclasine (1 μM) (Ye et al., 2017; Akkireddy et al., 2022; Wang et al., 2022) was loaded into the donor chamber of two wells of the RED insert in the 96-well dialysis plate. Blank phosphate-buffered saline (PBS) (500 μL) was added to each corresponding receiver chamber. After 5 h of incubation (with shaking at 37 °C), both sides were aliquoted, and the matrix was normalized. Samples were centrifuged at 1000 *g* for 10 min after adding five volumes of acetonitrile with IS. Supernatants were diluted 1:1 with water and analyzed with LC-MS/MS. Plasma Protein binding was calculated as follows:

% Bound = ((PAR in Donor–PAR in Receiver)/(PAR in Donor)) × 100; PAR = peak area ratio to an internal standard, including applicable dilution factors.

### 2.9 Biotransformation metabolite ID using hepatocytes

The above Hepatocyte experiment generated metabolite samples and performed ultra-high–pressure LC Tandem Mass Spectrometry (QTrap 6500+) for Metabolite ID Profiling. Mass spectrometric conditions were adapted from previously described methods (Heinonen et al., 2012; Xiao et al., 2012; Ballard et al., 2020). Briefly, reconstituted samples were analyzed by UHPLC-UV-MS operated in positive ion mode using a Qtrap mass spectrometer. For UHPLC-UV-MS analysis, the curtain gas (CUR) is 38, the Collision Gas (CAD) is medium, the Ion spray voltage (IS) is 5500 V, the Ion source gas (GS1) is 60, the Ion source gas (GS2): 65 and source heater was set at temperature: 600 °C source potential was 5500 V positive mode scanning. The mass spectrometer was operated in an EMS-EPI, MRM-EPI, and MRM mode. The normalized collision energy for the EPI scans was 20–45 V. Zorbax SB-C8,3.5 µm 4.6*150 mm column (Agilent, Santa Clara, CA) was used with a flow rate of 0.4 mL/min at 35 ^°^C. Mobile phase A was comprised of 5 mM Ammonium acetate, and mobile phase B was comprised of methanol. The gradient system used was as follows: 95% aqueous/5% organic for 0.0–0.5 min, then a linear gradient to 5% aqueous/95% organic at 14 min hold for 16.0 min, then bring back to 95% aqueous/5% organic at 17 min, hold the same up to 20 min.

### 2.10 Inhibition of CYP enzymes by narciclasine

Chemotherapy is often administered in combination with other drugs. To determine the inhibition of CYP enzymes by narciclasine, the samples were preincubated for 15 min at 37 °C in a shaking water bath (Valicherla et al., 2019; Zhou et al., 2020). The reaction was initiated by adding NADPH, and incubation proceeded for 10 min for CYP1A2 and CYP2C9 and 15 min for CYP2D6 and CYP3A4. The reaction was terminated by adding 200 µL of ice-cold acetonitrile containing the internal standard. Samples were vortexed for 5 min at 900 rpm using a Plate Mixer (Mix Mate) and then centrifuged for 10 min at 3,300 rcf to pellet the precipitated protein. The 50 µL supernatant was transferred to 96-well plates containing 100 µL of water, and the plates were vortexed for 1 min at 900 rpm. Finally, the samples were analyzed using LC-MS/MS with an XBridge C18 3.5 µm 2.1 × 50 mm (Waters Corporation, Milford, MA) column and a 3 mM ammonium acetate mobile phase with gradient flow at 0.4 mL/min.

### 2.11 Caco-2 permeability

Caco-2 (purchased from American Type Culture Collection) cells were used to model human intestinal drug absorption as described previously (van Breemen and Li, 2005; Patil et al., 2010) using 2 μM of narciclasine. Cell monolayers were dosed on the apical side (A to B: apical to basolateral permeability) or basolateral side (B to A: basolateral to apical permeability) and incubated at 37 °C with 5% CO2 in a humidified incubator. Samples were collected from the donor and receiver chambers at 2 h. Each experiment was performed in triplicate. All samples were analyzed by LC-MS/MS. The apparent permeability (Papp) and percent recovery were calculated as described previously (Patil et al., 2010).

### 2.12 Immunoblotting

Protein concentration was determined by the BCA assay. A total of 20 μg of protein lysate was separated on NuPAGE 4%–12% SDS-PAGE gradient gels and transferred to a nitrocellulose membrane (Cytiva) using a TE 70 semi-dry transfer unit (GE Healthcare). The membrane was then blocked with 1% BSA and then incubated with the indicated primary antibodies at 1:1,000, anti-MDR1 (Cat#13342s, RRID: AB_2631176) and anti-GAPDH (Cat#sc-47724, RRID: AB_627678), both diluted at 1:1,000. The membrane was then incubated with HRP-conjugated secondary antibodies, diluted 1:3,000, followed by detection with enhanced chemiluminescence.

### 2.13 *In vitro* cytotoxicity assay

Ovarian cancer cells (3,000–5,000 cells per well) were seeded and incubated in 96-well plates for 24 h before treatment with Narciclasine (1 nM–10μM, in 3-fold increments). Each drug concentration was used in six replicates. After 48 h of drug incubation, cell viability was measured using the DIMSCAN assay.

### 2.14 Exogenous expression of genes by transduction

HEK-293FT cells were plated and incubated until 70% confluent. The cells were then co-transfected with either the lentiviral ABCB1 (RC216080L3) plasmid or the control (PS100092) plasmid, along with the Lenti-vpak Packaging kit (OriGene). The virus-containing medium was collected every 24 h for 72 h, filtered (0.45 μm), and used to infect A2780 and SK-OV-3 cells. The virus-infected stable clones were obtained after selection for up to 2 weeks with puromycin at 1.5 or 2 μg/mL. Protein expression in stable clones was confirmed by immunoblotting.

### 2.15 Annexin V/PI staining

0.8 × 10^6^ cells were seeded in a T25 flask and treated with either Vincristine or Narciclasine for 24 h the following day. Apoptosis was assessed using the FITC Annexin V Apoptosis Detection Kit (BD Biosciences) following the manufacturer’s protocol, with staining for FITC Annexin V and Propidium iodide. Data were acquired using BD LSRFortessa X-20 with FACSDiva™ and analyzed with FlowJo v10 (BD Biosciences).

### 2.16 Statistical analyses

Student’s t-test was used to determine statistically significant differences on MS Excel and GraphPad Prism. *P*-values were two sided and tests were considered significant at *p* < 0.05. All the experiments were performed in triplicate and were consistently repeatable; for simplicity, one representative experiment for each condition is shown.

## 3 Results

### 3.1 Assay validation

To measure narciclasine in different matrices, we developed and LC/MS/MS assay. The method was validated for selectivity, linearity, precision, accuracy, recovery, matrix effect, dilution integrity, and stability in accordance with FDA guidance on bioanalytical method validation (Food and Drug Administration, 2018). Selectivity was assessed by analyzing six blank plasma lots for interfering peaks at the retention times of the analytes and internal standard (IS). No interfering peaks were detected, confirming acceptable selectivity. Carryover was evaluated by injecting a double-blank sample immediately after the upper limit of quantitation (ULOQ) sample.

Calibration curves were constructed by plotting the peak-area ratios (y) of the analytes to the IS against nominal concentrations (x) using a weighting factor of 1/x^2^. The curves were linear over the concentration range of 0.99–200 ng/mL, with a correlation coefficient (r) > 0.9984. The regression equation was y = 0.0144x + 0.00078 (r = 0.9984). The limit of detection (LOD) and lower limit of quantitation (LLOQ) were 0.2 ng/mL and 0.99 ng/mL, respectively. Intra- and inter-day accuracy and precision were determined using quality control (QC) samples at the LLOQ and at low, medium, and high concentration levels, as summarized in Table 1. Intra-day accuracy ranged from 88.15% to 109.33%, with precision <8.53%; inter-day accuracy ranged from 90.13% to 107.50%, with precision <8.23%. These results confirm that the method is accurate and precise, supporting its application to the determination of the ADME properties of narciclasine. Extraction recovery and matrix effect data for narciclasine and the IS are summarized in Table 2.

**TABLE 1:** Precision and accuracy for the analysis of narciclasine.

Nominal conc. (ng/mL)	Intra-assay (n=6)			Inter-assay (n=4)		
	Observed conc. (ng/mL)	Accuracy (%)	Precision (RSD%)	Observed conc. (ng/mL)	Accuracy (%)	Precision (RSD%)
1.08	1.02 ± 0.09	94.69	8.53	0.98 ± 0.05	90.78	4.79
3.11	2.95 ± 0.17	94.72	5.79	3.04 ± 0.25	97.78	8.23
96.00	104.96 ± 2.53	109.33	2.42	103.20 ± 1.14	107.50	1.11
160.00	141.04 ± 2.63	88.15	1.86	144.21 ± 2.19	90.13	1.52

**TABLE 2:** Recovery of narciclasine and internal standard (n = 6).

Nominal conc. (ng/mL)	Recovery		Internal standard recovery	
	Mean ± SD	Precision (RSD%)	Mean ± SD	Precision (RSD%)
3.11	106.63 ± 6.74	6.32	99.73 ± 2.10	2.1
96.00	107.54 ± 4.77	4.44	​	​
160.00	111.44 ± 6.74	3.43	​	​

Analyte stability in the matrix was assessed by analyzing QC samples stored under various conditions. Freeze/thaw stability, short-term stability, long-term stability, benchtop stability, and post-preparative stability results for narciclasine are presented in Table 3. Dilution integrity, which ensures accurate quantification of samples above the ULOQ, along with the corresponding accuracy and precision data, is summarized in Table 4.

**TABLE 3:** Stability of narciclasine in matrix at different conditions.

Storage conditions	Nominal conc. (ng/mL)	Mean ± SD	Precision (RSD%)
Freeze-thaw stability^a^	3.11	105.98 ± 0.19	5.88
	160.00	102.12 ± 1.54	0.94
Short-term stock stability^b^	3.11	101.41 ± 11.27	11.11
	160.00	100.49 ± 8.97	8.93
Long-term stability^c^	3.11	103.49 ± 0.27	8.42
	96.00	106.62 ± 4.56	4.46
	160.00	96.36 ± 12.65	8.20
Bench-top stability^d^	3.11	105.87 ± 0.10	3.19
	160.00	103.96 ± 2.03	1.22
Autosampler stability^e^	3.11	104.24 ± 0.22	6.90
	160.00	95.92 ± 12.77	8.32

^a^ Storage from −80 °C to 25 °C through three cycles.

^b^ Short term stock stability for 6 h.

^c^ Long term stability (4 days).

^d^ Bench top stability (4hrs).

^e^ Storage in processed samples in the autosampler at 4 °C for 24 h.

**TABLE 4:** Accuracy and precision of dilution integrity.

Dilution integrity (HQC fold dilution)	Nominal conc. (ng/mL)	Accuracy (%)	Precision (RSD%)
5	32.00	95.07 ± 2.40	7.9
10	16.00	102.71 ± 0.71	4.3

HQC: high quality control.

### 3.2 Solubility and lipophilicity

Solubility and lipophilicity are critical physicochemical properties in drug development, as they govern absorption and, consequently, therapeutic efficacy. Narciclasine demonstrated high kinetic and thermodynamic solubility (>400 µM), exceeding the preclinical benchmarks of >10 mM kinetic solubility and >50 mg/mL thermodynamic solubility (Sou and Bergström, 2018). Lipophilicity was assessed using the shake flask method (see Methods). The resulting LogD value of 0.40 (Table 5) indicates that narciclasine is predominantly hydrophilic, falling within the acceptable range of −1.5 to 4.5 but below the optimal range of 1–3 at pH 7.4 associated with sufficient membrane penetration. A LogD of 0.40 means narciclasine partitions 2.5-fold more into the organic phase than the aqueous phase, suggesting potentially limited cell permeability. Nevertheless, narciclasine exhibited IC50 values in the low nanomolar range across cancer cell lines, implying that enhanced membrane penetration could further increase its potency.

**TABLE 5:** Solubility and lipophilicity of narciclasine.

Experiment	Condition	Solubility (µM)	LogD
Thermodynamic solubility	PBS	453.32 ± 8.13	NA
Kinetic solubility	pH 5.0	439.8 ± 29.6	NA
	pH 7.4	449.0 ± 29.3	NA
	pH 10.0	454.1 ± 7.3	NA
Lipophilicity	pH7.4	NA	0.40 ± 0.01

NA: not applicable, LogD: logarithm of the distribution coefficient, values are mean ± SD.

### 3.3 *In vitro* stability in plasma, liver microsomes, and hepatocytes

**FIGURE 1:**
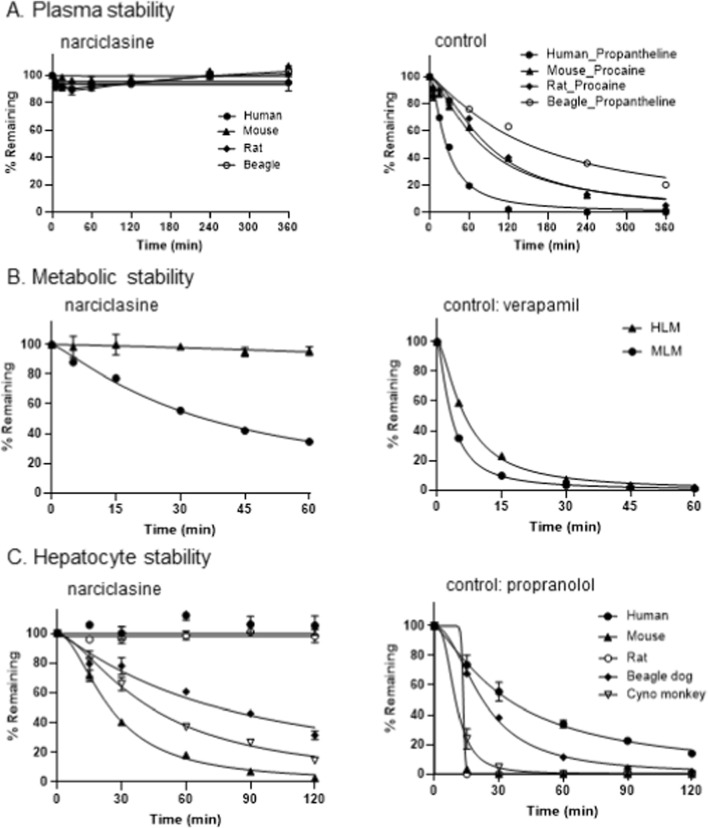
Plasma, metabolic, and hepatocyte stability of narciclasine. **(A)** The plasma stability of narciclasine was evaluated in plasma from four species, including humans (black circles), mice (black triangles), rats (black diamonds), and dogs (white circles) (left). The right panel is the positive control. **(B)** The metabolic stability of narciclasine was assessed in human liver microsomes (HLM, black triangles) and mouse liver microsomes (MLM, black circles) and presented on the left panel. The right panel is the positive control using verapamil. **(C)** The hepatocytic stability of narciclasine was tested in five species, including (black circles), mouse (black triangles), rat (white circles), beagle dog (black diamonds), and cynomolgus monkey (cyno monkey, white reverse triangles). The right panel is a positive control.

The plasma stability of narciclasine was assessed across four species: human, beagle dog, Sprague-Dawley (SD) rat, and mouse. This multi-species approach is essential to maximize the translatability of preclinical safety and efficacy data to humans, ensure regulatory compliance, and select the best species for definitive toxicology studies. The differences and similarities of metabolic stability are informative in interpreting preclinical data and in anticipating the responses in humans. At a concentration of 1 μM, narciclasine remained stable throughout 6 h of incubation in plasma from all four species, with no measurable decrease in concentration (Table 6; Figure 1A). The 6-h incubation period was chosen to confirm compound stability over the duration of the subsequent plasma protein binding assay, which required 5 h. In plasma protein binding experiments, the unbound fraction of narciclasine was highest in mice (61.5% ± 1.57%) and lowest in humans (43.7% ± 0.07%). The relatively low unbound fraction in humans suggests extensive protein binding, which may slow tissue distribution and reduce the free drug available for metabolism and elimination. This could explain the discrepancy between narciclasine’s excellent *in vitro* cytotoxicity and its moderate *in vivo* activity, potentially reflecting a shorter effective residence time in systemic circulation.

**TABLE 6:** Plasma stability and protein binding of narciclasine.

Experiment	Human	Dog	SD rat	Mice
Plasma stability	​	​	​	​
% Remaining at 360 min	94.7 ± 6.16	102.9 ± 3.84	100.7 ± 1.66	106.8 ± 0.64
t1/2 (min)	>360	>360	>360	>360
Plasma protein binding (fu)	43.7 ± 0.07	50.9 ± 3.0	58.8 ± 1.79	61.5 ± 1.57

t1/2: half-life, fu: % unbound fraction.

Next, we tested microsomal stability in two species, human and mouse (Table 7; Figure 1B). In human liver microsomes, >87% (with 5.1% SD) of narciclasine was detected at 60 min of incubation, with a half-life of >120 min. This indicates that narciclasine is stable in human liver microsomes. On the other hand, approximately half (34.47% ± 0.29%) of narciclasine was recovered at 60 min in mouse liver microsomes. These data show a difference in microsomal stability between humans and mice, indicating that narciclasine is minimally metabolized in the liver and suggesting that the majority of the compound will be eliminated via the kidney.

**TABLE 7:** Microsomal and hepatocyte stability of narciclasine.

Species	Parameters	Metabolic stability (60 min)	Hepatocyte stability (120 min)
Human	% remaining	87.34 ± 5.07	105.03 ± 6.37
	t1/2 (min)	>60	>120
	CL_int_ (µL/min/mg protein)	1.43 ± 1.18	NA
	CL_int_ (µL/min/10^6^ cells)	NA	0.38 ± 0.24
CD1 mice	% remaining	34.47 ± 0.29	2.72 ± 0.32
	t1/2 (min)	NA	22.99 ± 0.72
	CL_int_ (µL/min/mg protein)	36.03 ± 1.17	NA
	CL_int_ (µL/min/10^6^ cells)	19.25 ± 0.61	30.16 ± 0.94
Beagle dog	% remaining at 120 min	ND	98.08 ± 4.37
	t1/2 (min)	ND	>120
	CL_int_ (µL/min/10^6^ cells)	ND	0.21 ± 0.2
SD rat	% remaining at 120 min	ND	31.62 ± 2.94
	t1/2 (min)	ND	77.16 ± 4.54
	CL_int_ (µL/min/10^6^ cells)	ND	9.00 ± 0.52
Cyno monkey	% remaining at 120 min	ND	18.99 ± 1.30
	t1/2 (min)	ND	47.17 ± 1.54
	CL_int_ (µL/min/10^6^ cells)	ND	14.70 ± 0.49

t1/2: half-life, NA: not applicable, ND: not determined.

Hepatocyte stability of narciclasine was evaluated across five species: human, dog, rat, mouse, and monkey (Table 7; Figure 1C). Propranolol and umbelliferone were included as positive controls to confirm that the hepatocytes were metabolically active. After 120 min of incubation, narciclasine concentrations remained stable in human and dog hepatocytes, indicating low hepatic clearance in these species. In contrast, narciclasine was more rapidly metabolized in rat, mouse, and monkey hepatocytes, with half-lives of 77, 23, and 47 min, respectively, corresponding to moderate clearance in rat and high clearance in mouse and monkey. These findings are consistent with microsomal stability data, collectively indicating that hepatic metabolism of narciclasine is minimal in humans and dogs but substantially higher in rodents and monkeys.

### 3.4 Identification of narciclasine metabolites

**FIGURE 2:**
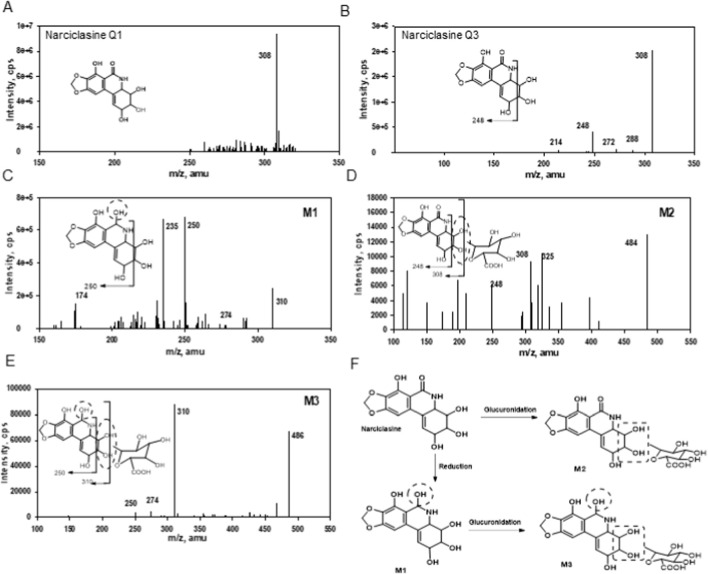
Identification of tentative metabolites and metabolic pathways of narciclasine in human liver microsomes. **(A)** The Q1 mass spectrum of narciclasine is presented. **(B)** Q3 mass spectrum of narciclasine shows a major fragment ion, m/z 248. **(C–E)** Structural identification of M1-M3 using tandem mass spectrometry. Tentatively, the reduction occurred at the C6 ketone position (M1), and glucuronidation occurred on one of the two hydroxyl groups, as shown in the dotted circle (M2). Also, M3 with both reduction and glucuronidation was detected. **(F)** Proposed phase I and II metabolic biotransformation pathways of narciclasine are presented.

Using LC/MS/MS, we identified the metabolites of narciclasine after incubating in hepatocytes of humans, mice, rats, dogs, and monkeys (Figure 2). The purpose of the experiments is to determine the sites and the types of biotransformation of nartciclasine. As shown in Figure 2, the biotransformation of narciclasine (Figures 1B,C) was minimal in human liver microsomes and hepatocytes, and we detected only one tentative metabolite (M1) in Human hepatocytes which was also present in four other species (Figure 2C). The metabolite was detected using a positive precursor ion scan at m/z 310, which occurs after the reduction of the C6 carbon. Two additional tentative metabolites, M2 and M3, were identified in all four other species except humans (Figures 2D,E). M2 and M3 showed [M + H]+ ions at m/z 484 and 486, and the fragmentation spectra corresponding to the direct addition of glucuronide (176 amu) and the reduction followed by glucuronidation to narciclasine. A proposed biotransformation pathway for narciclasine is shown in Figure 2F, based on data collected from hepatocytes of different species.

### 3.5 Enzymatic inhibition of narciclasine against cytochrome P450

**FIGURE 3:**
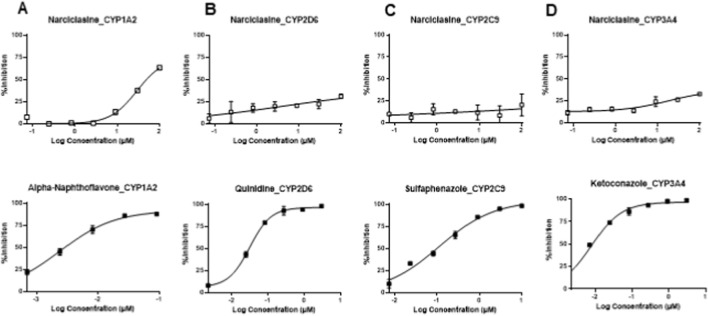
Inhibition of CYP enzymes by narciclasine. The inhibitory effect of narciclasine on the CYP1A2 **(A)**, CYP2D6 **(B)**, CYP2C9 **(C)**, and CYP3A4 **(D)** isoforms was evaluated. The substrates and the concentrations used for each isoform of CYP450 are listed in Supplementary Table S3, and the mass transition of the metabolites of the substrates and the internal standard are provided in Supplementary Table S4. The top panel is for narciclasine’s inhibitory effect on each isoform of CYP enzyme, and the bottom panel is for the positive controls to confirm the inhibitory effect on the corresponding isoforms.

Next, we investigate the inhibitory potential of narciclasine against CYP1A2, CYP2C9, CYP2D6, and CYP3A4. These experiments are to examine potential drug-drug interactions of narciclasine with comcommitant treatment. In the experiments, a concentration range of 0.1 μM–100 μM was used. In human liver microsomes, we incubated individual substrates (diclofenac for 1A2, dextromethorphan for 2C9, phenacetin for 2D6, and verapamil for CYP3A4) with narciclasine or positive control (α-naphthoflavone for CYP1A2, quinidine for CYP2D6, sulfaphenazole for CYP2C9, and ketoconazole for CYP3A4). We measured the formation of metabolites (Supplementary Tables S2, S3) and their mass transitions (Supplementary Table S4). Narciclasine exhibited a weak inhibitory effect on CYP1A2, with an IC_50_ value of 54.90 μM, while the IC50 values for CYP2C9, CYP2D6, and CYP3A4 (Figures 3A–D) were over 100 μM. These data indicate that narciclasine may interact with drugs that are metabolized by CYP1A2 but do not interfere with the metabolism of the drugs metabolized by CYP2C9, CYP2D6, and CYP3A4.

### 3.6 P-glycoprotein substrate status of narciclasine

There are many known P-glycoprotein substrates that are indicated for various disease. In early stages of development, it is informative to get the agnet tested to determine the status of being P-glycoprotein substrates. We used two independent experiments to evaluate whether narciclasine is a P-glycoprotein substrate: 1) Caco-2 permeability assay and 2) determining cytotoxic activity in human cancer cells with exogenous expression of p-glycoprotein. To measure the absorption of narciclasine, we used the Caco-2 cell permeability assay, which assesses efflux ratios (Papp A to B/Papp B to A). The cells were cultured for 21 days as a monolayer before transporter inhibitors, positive control, and narciclasine were added to the culture. The average efflux ratio for digoxin, the ratio of the permeability of digoxin moving from the basolateral to the apical side (efflux) compared to the permeability moving from the apical to the basolateral side (absorption), was 6.9 in Caco-2 when 2 μM narciclasine was used. The specific concentration was selected after testing 1, 2, 2.5, and 10 μM of narciclasine in preliminary experiments: 1 μM did not produce a detectable level of narciclasine in receiver samples, and 2.5 and 10 μM affected the viability of Caco-2 cells. Narciclasine recovery was within the acceptable range (90%–98%) across all efflux transporter assays. In Caco-2 cells, the apparent permeability from apical to basolateral side (Papp A to B) of narciclasine was 4.49 × 10^−6^ cm/s on average from two independent measurements. The permeability measured in the opposite direction (Papp B to A) was 5.39 × 10^−6^ cm/s, indicating that narciclasine has low permeability.

**FIGURE 4:**
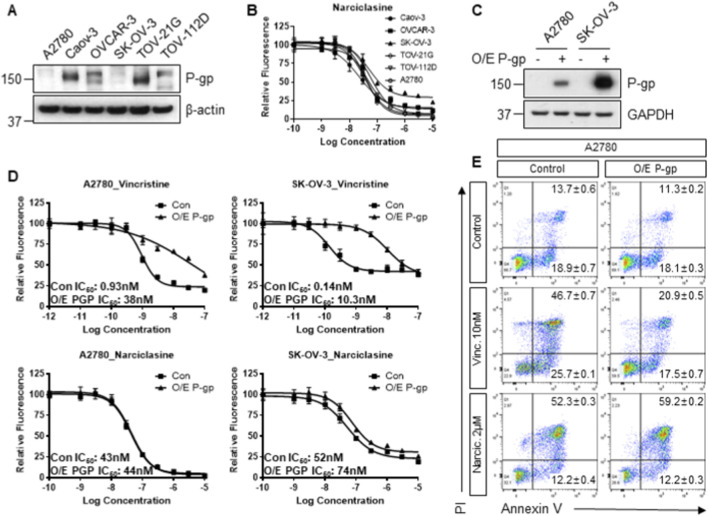
Narciclasine sensitivity in P-glycoprotein-overexpressing ovarian cancer cells. **(A)** The constitutive expression levels of P-glycoprotein were evaluated in six ovarian cancer cells by immunoblotting. β-actin was used as a loading control. **(B)** The dose-response curves of ovarian cell lines to narciclasine (1nM–10 µM) with 24 h incubation are presented. Data represent the mean ± SD of six replicates. **(C)** The exogenous expression of P-glycoprotein was confirmed in two isogenic pairs of ovarian cancer cell lines, A2780 and SK-OV-3, by immunoblotting. GAPDH was used as a loading control. **(D)** The dose-response curves of the two isogenic pairs with or without exogenous expression of P-glycoprotein to vincristine (0.01nM–100 nM) or narciclasine, incubated for 24 h, are presented. **(E)** Representative cytograms showing apoptosis in A2780 treated with 10 nM Vincristine or 2 µM narciclasine for 24 h are shown. Data represent the mean ± SD of triplicates. All control samples were treated with 0.1% DMSO as a vehicle control.

To compare the extent of the P-gp effect in the anti-tumor cytotoxicity of narciclasine, we evaluated the P-gp expression and narciclasine cytotoxicity in seven ovarian cancer cell lines (Figures 4A,B). The IC_50_ values of narciclasine ranged from 23 to 58 nM, with SK-OV-3 and TOV-112D having the two highest values (52 and 58 nM, respectively). Then, we exogenously expressed P-gp in the 2 cell lines with low P-gp expression, A2780 and SK-OV-3 (Figure 4C), and compared the cytotoxicity of narciclasine using vincristine (Souza et al., 2011) as a positive control (Figure 4D). While vincristine exhibited 40–100-fold differences in IC_50_ values in cells with P-gp overexpression relative to the control, narciclasine showed minimal IC_50_ differences between the two conditions. We also assessed changes in anti-tumor activity in cell lines with constitutively high P-gp expression, Caov-3 and TOV-21G, using verapamil, a P-gp inhibitor (Wang and Sun, 2020). When cells were co-incubated with a non-toxic concentration of verapamil, vincristine cytotoxicity was significantly affected, but narciclasine dose-response curves and narciclasine + verapamil superimposed (Supplementary Figures S1A,B). Lastly, the percentage of apoptotic cells treated with vincristine was significantly lower in P-gp-overexpressing cells (p < *0.001* for both). However, minimal differences in cell morphology and % apoptotic cells were observed in narciclasine-treated cells (p = 0.829; Figure 4E; Supplementary Figures S1C,D) relative to untreated controls. These data indicate that narciclasine is not a P-gp substrate.

## 4 Discussion

Narciclasine has been investigated by multiple research groups as a potential cancer chemotherapeutic. It exhibits excellent *in vitro* cytotoxic activity at low nanomolar concentrations across a range of cancer types, and its efficacy in *in vivo* xenograft models has been significant in some xenograft models of human cancers while the activity in other histology is less (Wei et al., 2023). In the current study, we attempted to provide comprehensive data on the pharmacological properties of narciclasine, including stability, solubility, metabolism, and P-glycoprotein substrate status which may help further development of narciclasine.

Aqueous stability and lipophilicity experiments confirm that narciclasine is predominantly hydrophilic, which presents known challenges for drug delivery, particularly with respect to cell membrane penetration given the lipid bilayer barrier (Rao et al., 2023). However, the molecular weight of narciclasine is 307.26 Da, well below the 500 Da threshold for oral absorption and cell permeability, which may partially offset its hydrophilicity. Consistent with this, narciclasine retains nanomolar cytotoxicity, suggesting that relatively low intracellular concentrations are sufficient to kill cancer cells (Wei et al., 2023). Low albumin binding is an additional factor that may reduce the systemic residence time of narciclasine. *In vivo* pharmacokinetic (PK) studies have reported a mean residence time (MRT0→ ∞ ) of approximately 3 h (Chen et al., 2021; Ren et al., 2021), which may contribute to its modest *in vivo* efficacy despite favorable potency.

Metabolic stability data demonstrate that narciclasine undergoes species-dependent metabolism. In human liver microsomes, metabolic transformation was minimal, with only a small fraction of narciclasine undergoing reduction, glucuronidation, or both. Metabolism was more extensive in mouse liver microsomes; however, more than 30% of the compound remained unchanged after 60 min, indicating that narciclasine is not extensively metabolized by the liver in any species tested. Hepatocyte stability experiments yielded consistent results: narciclasine remained stable in human and dog hepatocytes but underwent partial transformation in rat, monkey, and mouse hepatocytes. Notably, tentative metabolites exhibited significantly higher IC50 values than the parent compound, suggesting that metabolism contributes to the inactivation of narciclasine (Ingrassia et al., 2009). These findings have important implications for the translational development of narciclasine. Specifically, the metabolic and pharmacokinetic profile observed in xenograft mouse models may not accurately predict behavior in humans. In humans, narciclasine appears to be primarily eliminated via the renal route, which may reduce systemic exposure and limit *in vivo* efficacy. To address these limitations, two strategies warrant consideration. First, structural modifications to develop prodrugs could reduce renal elimination and improve lipophilicity by selecting analogs with higher LogD values. Second, liposomal or nanoparticle-based formulations could extend the mean residence time (MRT) of narciclasine, though hepatic accumulation should be monitored in such approaches. Together, these strategies could meaningfully enhance the *in vivo* pharmacological activity of narciclasine.

Caco-2 permeability experiments indicate that narciclasine is not a substrate of P-glycoprotein (P-gp), notwithstanding the potential limitations to cell penetration posed by its hydrophilicity and low molecular weight (Supe and Takudage, 2021). To further investigate the role of P-gp, we exogenously expressed P-gp in an ovarian cancer cell line and assessed its effect on narciclasine’s cytotoxic activity. The results confirm that narciclasine’s *in vitro* cytotoxicity is minimally affected by P-gp expression. This is a particularly favorable characteristic, as many natural product-derived chemotherapeutics are P-gp substrates and therefore susceptible to efflux-mediated resistance (Iqbal et al., 2022). The absence of P-gp-mediated efflux thus represents a potential advantage for the future clinical development of narciclasine.

In conclusion, narciclasine is a small molecule under preclinical evaluation for development as an anticancer agent. The current study developed a robust LC/MS/MS assay to detect narciclasine in biological matrices and provided a comprehensive characterization of its pharmacological properties, including stability, metabolism, metabolite identification, and permeability. We further demonstrated that the *in vitro* cytotoxic activity of narciclasine is not compromised by P-gp expression. The data provided in this study will improve the pharmacological understanding of narciclasine, which is needed for further development of the agent.

## Data Availability

The original contributions presented in the study are included in the article/Supplementary Material, further inquiries can be directed to the corresponding author.
