# Supplementary material for: In Vitro characterization of narciclasine: solubility, metabolic stability, and P-glycoprotein substrate status
Source: Front Pharmacol. 2026 Apr 28;17:1768477. doi: 10.3389/fphar.2026.1768477 (PMC13161073; doi:10.3389/fphar.2026.1768477)
Supplement: Supplementary file 1 [file Supplementaryfile1.docx]

**Supplemental Methods**

*Sample Preparation*

1. Plasma Protein Binding:

The samples were matrix-matched with the opposite matrix (at the end of incubation, 25 µL of plasma-donor sample with 25 µL of buffer; similarly, 25 µL of buffer-receiver sample with 25 µL of plasma). Matrix-matched samples were precipitated with 300 µL of acetonitrile containing an internal standard, vortexed for 5 min at 900 rpm, centrifuged at 3300 rcf for 10 min, and the supernatant was diluted with an equal volume of water and analyzed by LC-MS/MS.

1. Metabolic Stability:

25 µL of the incubation mixture will be quenched with 300 µL of ice-cold acetonitrile containing the internal standard at different time points (5, 15, 30, 45, and 60min). All the quenched samples were preserved at 2-8°C until the experiment, then vortexed for 5 min at 900 rpm, centrifuged at 3300 rcf for 10 min, the supernatant diluted with an equal volume of water, and analyzed by LC-MS/MS.

1. Hepatocyte Stability:

At each time point (0, 15, 30, 60, 90, 120 min), 25 µL of the incubation mixture was precipitated with 200 µL of acetonitrile containing the internal standard. All the quenched samples were preserved at 2-8°C until the completion of the experiment, then vortexed for 5 min at 900 rpm, centrifuged at 3300 rcf for 10 min, the supernatant diluted with an equal volume of water, and analyzed by LC-MS/MS.

1. Plasma stability:

At each time point (0, 5, 15, 30, 60, 120, 240, and 360 min), 25 µL of the incubation mixture was precipitated with 200 µL of acetonitrile containing the internal standard. All the quenched samples will be preserved at 2-8°C until the experiment. Then, the samples were mixed for 5 min at 900 rpm, centrifuged at 3300 rcf for 10 min, the supernatant diluted with an equal volume of water, and analyzed by LC-MS/MS. The stability of analytes in the matrix was tested by analyzing QC samples stored under different conditions. Freeze/thaw stability was assessed after three cycles of thawing at room temperature and freezing at −80 °C. Short-term stability was assessed after 6 h at room temperature (25 °C). The long-term stability was evaluated after keeping at −80 °C for 3 days. Benchtop stability was assessed after 4h at room temperature. The post-preparative stability was determined by analyzing QC samples after storage in the autosampler at 4 °C for 24 h.

1. Caco2 sample preparation:

At the end of the incubation, 25 µL of assay samples were precipitated with 300 µL of acetonitrile containing internal standard (Clofarabine), vortexed for 5 mins at 900 rpm, and centrifuged at 3300 rcf for 10 min. After centrifugation, 150 µL of the supernatant was diluted with 150 µL of Milli-Q water and analyzed by LC-MS/MS.

1. CYP Inhibition sample preparation:

The incubation mixtures were composed of 0.2 mg/mL microsomal protein, 1 mM NADPH, 100 mM potassium phosphate buffer (pH 7.4), and individual substrates (Diclofenac, Dextromethorphan, Phenacetin, and Verapamil) in a total volume of 200 µL. The potassium phosphate buffer was freshly prepared by dissolving 13.98 g of dibasic potassium phosphate and 2.7 g of monobasic potassium phosphate in 1000 mL of water, and the pH was adjusted to 7.4. The substrates were used at concentrations corresponding to their respective Km values: 5 µM for Diclofenac, 10 µM for Dextromethorphan, 70 µM for Phenacetin, and 1 µM Verapamil. The final solvent concentration was less than 1.0%.

*Metabolic Stability and inhibition of metabolism*

In the microsomal stability experiment, each reaction mixture (250 μL) consisted of narciclasine (1 μM), human and CD-1 mouse (Life Technologies Corporation, USA, CA) microsomal fractions (1.0 mg/mL), and NADPH (1 mM) in phosphate buffer at pH 7.4. Reaction mixtures were incubated in 96-well plates at 37°C, and time points were collected serially at 0, 5, 10, 15, 30, 45, and 60 minutes. The reaction was terminated by adding five volumes of cold acetonitrile with an internal standard (IS). Samples were analyzed using UPLC-MS/MS. Clearance (Clint) and half-life calculations were performed. In hepatocytes, a Cryopreserved mixed-gender human pool of 10 male CD-1 mice, male hepatocytes (Life Technologies Corporation, CA, USA), was thawed, equilibrated at 37°C, and 200 µL of 2 million cells was seeded in a culture plate (Celltreat, 48-well plate) per well. The hepatocytes were pre-incubated for 10 minutes. The reaction was then initiated by adding 200 µL of a 2 µM working stock solution of the compounds, resulting in a final concentration of 1 µM narciclasine and 1 million hypocellularity. The reaction mixture was incubated at 37°C under 20% O2/5% CO2, with 95% relative humidity and shaking at 600 rpm. Aliquots were withdrawn at 0, 15, 30, 60, 90, and 120 minutes. The reaction was terminated by adding five volumes of cold acetonitrile containing the internal standard (IS). Samples were then centrifuged and analyzed by UPLC-MS/MS. 25 µL of the incubation mixture will be quenched with 300 µL of ice-cold acetonitrile containing the internal standard at different time points (5, 15, 30, 45, and 60 min). All the quenched samples were preserved at 2-8°C until the experiment was complete. Then, the samples were vortexed for 5 min at 900 rpm, centrifuged at 3300 rcf for 10 min, and the supernatant was diluted 1:1 with water before LC-MS/MS analysis.

**Supplemental Table 1.** Reagents and Chemicals used in this project.

| **Reagents / Chemicals** | **Catalog no.** | **Vendor** |
| --- | --- | --- |
| Narciclasine | N/A | Aktin Chemicals, Inc, China |
| Acetonitrile | 9829-03 | Avantor, Visalia, CA, USA |
| Methanol | BDH85800-400 | Avantor, Visalia, CA, USA |
| Procaine hydrochloride | PHR1161-1G | Sigma Aldrich Scientific, St. Louis, MO, USA |
| Propantheline HBr | PHR1575-500MG | Sigma Aldrich Scientific, St. Louis, MO, USA |
| Ketoconazole | PHR1385-1G | Sigma Aldrich Scientific, St. Louis, MO, USA |
| Ammonium formate | 17843-50G | Sigma Aldrich Scientific, St. Louis, MO, USA |
| Potassium phosphate monobasic | PX1562-5 | Sigma Aldrich Scientific, St. Louis, MO, USA |
| Potassium phosphate dibasic | p3786-100g | Sigma Aldrich Scientific, St. Louis, MO, USA |
| Propranolol HCl | P0884-1G | Sigma Aldrich Scientific, St. Louis, MO, USA |
| Diclofenac sodium | PHR1144-1G | Sigma Aldrich Scientific, St. Louis, MO, USA |
| Warfarin Sodium | PHR1435-1G | Sigma Aldrich Scientific, St. Louis, MO, USA |
| Dextromethorphan hydrobromide | D9684-5G | Sigma Aldrich Scientific, St. Louis, MO, USA |
| α-naphthoflavone | N5757-1G | Sigma Aldrich Scientific, St. Louis, MO, USA |
| Sulfaphenazole | 77668-14 | Avantor, Visalia, CA, USA |
| Phenacetin | 77440-50G | Sigma Aldrich Scientific, St. Louis, MO, USA |
| Elacridar | SML0486-10MG | Sigma Aldrich Scientific, St. Louis, MO, USA |
| Clofarabine | C7495-25mg | Sigma Aldrich Scientific, St. Louis, MO, USA |
| 1-Octanol | 472328-1L | Sigma Aldrich Scientific, St. Louis, MO, USA |
| SD rat plasma | 45162 | Innovative Research (Sarasota, Florida, USA) |
| CD1 mouse plasma | 45181 | Innovative Research (Sarasota, Florida, USA) |
| Human plasma K2EDTA male pooled | HMN1318061K | Bioivt (Hicksville, NY, USA), |
| Beagle plasma K2EDTA Gender Pooled | BGL158515 | Bioivt (Hicksville, NY, USA), |
| Digoxin | 228120010 | Thermofisher Scientific-Waltham, MA, USA |
| Atenolol | 449550010 | Thermofisher Scientific-Waltham, MA, USA |
| Verapamil hydrochloride | 329330010 | Thermofisher Scientific-Waltham, MA, USA |
| Mouse CD1 pooled liver microsomes | MSMPCL | Thermofisher Scientific-Waltham, MA, USA |
| Human liver microsomes 50 donor pool | HMMCPL | Thermofisher Scientific-Waltham, MA, USA |
| Mouse CD1 cryo hep male suspen | MSCS10 | Thermofisher Scientific-Waltham, MA, USA |
| Human cryo hep suspension pool | HMCS10 | Thermofisher Scientific-Waltham, MA, USA |
| Suspension pool Beagle cryo Hep male suspension | DGCS10 | Thermofisher Scientific-Waltham, MA, USA |
| Cyno cryo Hep male suspension | MKCS10 | Thermofisher Scientific-Waltham, MA, USA |
| Rat cryo Hep male suspension | RTCS10 | Thermofisher Scientific-Waltham, MA, USA |
| Hepatocyte thaw media | CM7500 | Thermofisher Scientific-Waltham, MA, USA |
| Williams Medium E | A12176-01 | Thermofisher Scientific-Waltham, MA, USA |
| Lucifer yellow (L453) | 11140050 | Thermofisher Scientific-Waltham, MA, USA |
| FBS | 10082147 | Thermofisher Scientific-Waltham, MA, USA |
| RED Device Inserts | 89809 | Thermofisher Scientific-Waltham, MA, USA |
| Reusable base plate | 89811 | Thermofisher Scientific-Waltham, MA, USA |
| Caco-2 cells | HTB-37 | ATCC, Manassas, VA USA |
| EMEM | 30-2003 | ATCC, Manassas, VA USA |
| TRYPSIN-1X | 0-2101 | ATCC, Manassas, VA USA |
| NADPH Tetra sodium Salt | 481973-50mg | EMD Millipore Corp-USA |
| 48 well tissue culture plates | 229147 | Celltreat scientific products, Pepperell MA, USA |
| Deep well 96 well plates | EP951031887-40EA | Eppendorf, Hamburg, Germany |
| Phosphate Buffered Saline | SH30028.02 | Cytica, HyClone Laboratories, Utah, USA |
| Permeable Support with 0.4 µm Pore Polycarbonate Membrane plates | 3391 | Corning, NY, USA |
| Estrone- 3 Sulfate Sodium salt | E0251-100mg | Sigma Aldrich Scientific, St. Louis, MO, USA |
| KO-143 hydrate | K2144-1mg | Sigma Aldrich Scientific, St. Louis, MO, USA |
| 7-Hydroxy coumarin | H24003-10G | Sigma Aldrich Scientific, St. Louis, MO, USA |

**Supplemental Table 2**. Permeability of narciclasine in CaCO2 cells.

| Inhibitor | Papp A to B (10^-6^ cm/s) | Papp B to A (10^-6^ cm/s) | Efflux ratio |
| --- | --- | --- | --- |
| without | 0.73 ± 0.12 | 5.09 ± 0.11 | 7.1 |
| Elacridar | 1.42 | 5.8 | 4.9 |

Active efflux of narciclasine (2 μM) in Caco-2 cell monolayers with or without elacridar (Pgp inhibitor).

**Supplemental Table 3**. Permeability of narciclasine in CaCO2 cells.

| CYP Isoform | Substrate | Metabolism | Metabolite | IC50 (μM) |
| --- | --- | --- | --- | --- |
| CYP1A2 | Phenacetin | O -Deethylation | Acetaminophen | 54.9 |
| CYP2D6 | Dextromethorphan | O -Demethylation | dextorphan | > 100 |
| CYP2C9 | Diclofenac | Hydroxylation | 4-hydrodiclofenac | > 100 |
| CYP3A4 | Verapamil | N -Demethylation | norverapamil | > 100 |

**Supplemental Table 4**. Transitions of metabolites and Internal standard.

| Compound | Parent (m/z) | Daughter (m/z) | Declustering potential | Collision (V) |
| --- | --- | --- | --- | --- |
| Phenacetin (CYP1A2) | 152 | 110.1 | 80 | 26 |
| Dextrormethorphan (CYP2D6) | 258.2 | 157.3 | 100 | 35 |
| Diclofenac (CYP2C9) | 312 | 229.8 | 80 | 36 |
| verapamil (CYP3A4) | 441.2 | 165.2 | 100 | 22 |
| Clofarabine (ISTD) | 304.2 | 170 | 100 | 29 |

**Supplemental Figure 1** Narciclasine activity concerning P-glycoprotein expression.

**
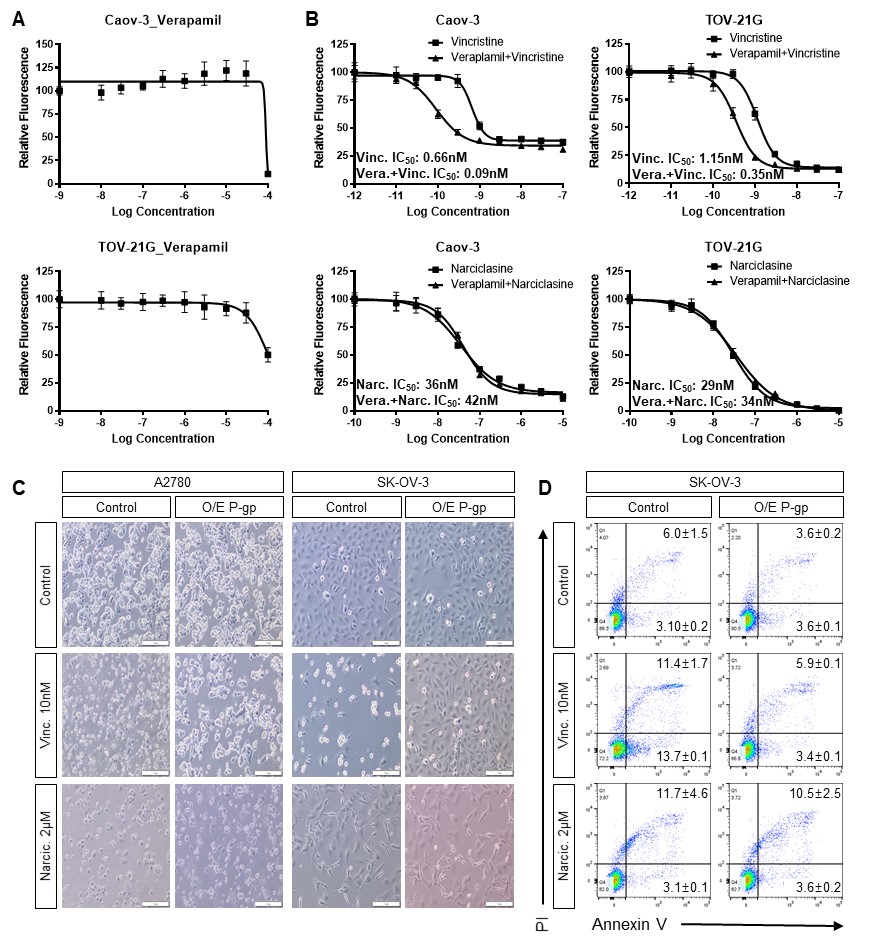
**

(A) Cells were treated with verapamil (10nM to 100µM at 3x concentrations) for 48 hours before the viability was assessed. (B) Both cell lines were pre-treated with verapamil (30µM or 20µM) for 1 hour, followed by vincristine or narciclasine at given concentrations. Vincristine was used as a positive control. (C) Representative morphology of cells treated with vincristine or narciclasine in cells transduced with a control vector or *MDR1* gene (10×). (D) Representative cytograms showing apoptosis in SK-OV-3 treated with 10nM Vincristine or 2µM Narciclasine for 24 hours. Data represent the mean ± SD of triplicates. All control samples were treated with 0.1% DMSO as a vehicle control.
